# Protocol for a process and implementation evaluation of the SMARThealth pregnancy hybrid type 2 cluster randomised controlled trial

**DOI:** 10.1177/17455057261425789

**Published:** 2026-03-02

**Authors:** Nicole Votruba, Sreya Majumdar, Sudhir Thout Raj, Vaaruni Nayak, Ankita Sharma, David Peiris, Hueiming Liu, Varun Arora, Minakshi Verma, Mohammad Abdul Ameer, Devarsetty Praveen, Jane E. Hirst

**Affiliations:** 1 Nuffield Department of Women’s & Reproductive Health, University of Oxford, UK; 2 The George Institute for Global Health, Imperial College, London, UK; 3 The George Institute for Global Health, New Delhi, India; 4 The George Institute for Global Health, University of New South Wales, Australia; 5 Faculty of Medicine and Health, University of Sydney, NSW, Australia; 6 PGIMS Rohtak, Haryana, India; 7 University of New South Wales, Sydney, NSW, Australia; 8 Prasanna School of Public Health, Manipal Academy of Higher Education, India

**Keywords:** maternal health, mHealth, non-communicable diseases, anaemia, gestational diabetes, preeclampsia, community health workers, rural India, complex intervention, clinical decision support

## Abstract

**Background:**

This protocol outlines the process and implementation evaluation of the SMARThealth Pregnancy (SHP) pragmatic, type 2 hybrid cluster randomised trial conducted in two states in India (Haryana/Telangana). The SHP trial aims to improve the community-level identification, diagnosis, referral and management of women with anaemia, diabetes, and hypertension during pregnancy and in the year after birth.

**Objectives:**

The process and implementation evaluation aims to understand how, why, and for whom the SHP intervention may be effective (or not). It aims to identify contextual factors, barriers, and facilitators relevant to the implementation of the intervention, and understand mechanisms and strategies employed during its implementation.

**Design:**

The study utilised a process evaluation design.

**Method:**

A mixed methods evaluation drawing from realist evaluation, normalisation process theory, the Medical Research Council framework, reach, effectiveness, adoption, implementation and maintenance framework, and Proctor’s typology will be employed for understanding the implementation process. The evaluation will involve focus group discussions and semi-structured interviews with healthcare providers (Accredited Social Health Activist, primary care doctors, and auxiliary nurse midwife), women, and field staff. Quantitative process data describing reach, fidelity, dose, and adoption of intervention will be collected. Observations of trial setup and implementation will be conducted. Both qualitative and quantitative data will be analysed iteratively before the effectiveness outcomes of the SHP trial are available and will subsequently be triangulated with the trial primary outcome evaluation data.

**Discussion:**

The findings from this process evaluation will provide an understanding of how the intervention works in practice, its potential to detect and manage anaemia, diabetes and hypertension during pregnancy and in the year after birth, and its scalability as an integrated model for the management of non-communicable diseases in pregnancy/postnatal care.

## Introduction

### Perinatal health and non-communicable diseases in India

Non-communicable diseases (NCDs) are women’s leading causes of death globally and in India.^1^ Women who develop complications during pregnancy are at increased risk for NCDs in the months and years following birth.^2^ Women with hypertensive disorders of pregnancy (HDP), such as gestational hypertension or preeclampsia, are at risk of later chronic hypertension and premature cardiovascular diseases,^3^ and those with gestational diabetes mellitus (GDM) are at a significantly increased risk of type 2 diabetes in years after birth.^4^ Although it has been recommended that pregnancy care should be part of an integrated approach to NCDs,^5–7^ clear evidence and guidance is missing on how this can best be performed.

In India, in addition to NCDs, anaemia rates amongst pregnant women are amongst the highest in the world. Anaemia is linked to poor pregnancy outcomes, such as post-partum haemorrhage, maternal death, low birth weight, preterm birth, and perinatal and neonatal mortality.^8^ The Indian Government has declared anaemia a health priority and launched the comprehensive national anaemia reduction programme (Anaemia Mukt Bharat) in 2018.^9^ Substantial progress has been made in maternal health; however, gaps in coverage and service delivery remain.^10^

Both NCDs and anaemia are leading challenges in maternal health in India, and their high prevalence has severe, lasting consequences for both mothers and their children. In addition, NCDs and anaemia are often interconnected and exacerbate one another, and both anaemia, HDP, and GDM have been identified as priority areas for improving maternal and child health in rural India.^11^

### Perinatal mobile health interventions

Mobile health interventions including clinical decision systems are increasingly being used in maternity care. Recent evaluations found improvements in safety and health outcomes; however, design of interventions, quality, complexity of systems, and evaluations were very heterogeneous.^12–14^ In particular, interventions that are working across ante- and postnatal stages, high-quality large-scale randomised controlled trials (RCTs), and evaluations that capture implementation outcomes of the intervention in routine primary care settings are rare.^15–17^

### The SMARThealth Pregnancy intervention

To fill this gap and in response to locally identified challenges around the timely detection and referral of high-risk conditions during and after pregnancy in rural India, we developed the SMARThealth Pregnancy (SHP) intervention.

SHP leverages the Systematic Medical Appraisal, Referral and Treatment (SMARThealth) platform, initially developed by the George Institute for Global Health for cardiovascular disease prevention and management.^18^ This platform is a mobile health clinical decision-support system designed to assist community health workers in screening various conditions using point of care testing and an app functioning via a low-cost Android tablet with clinical decision-support algorithms based on local guidelines. The app enables Accredited Social Health Activists (ASHAs) to electronically refer high-risk cases to the Primary Health Centre (PHC) for medical review. The primary care doctor also uses a connected tablet app for more complex decision support, such as medication management suggestions, based on national guidelines.^19–23^ The follow-up module of the platform supports the ASHAs to conduct timely follow-up visits and discuss relevant topics like visit to the doctor, adhering to healthy lifestyle and medications, or achieving target levels for BP and diabetes during the visit. SHP has adapted the SMARThealth approach for pregnant women to improve the identification, diagnosis, referral, follow-up and management of women with anaemia, diabetes, and hypertension during and after pregnancy.^24^ Acceptability and feasibility of SHP have previously been established in a pilot cluster randomised trial in four sites in rural India from 2019 to 2020.^25^

The SHP intervention comprises five main components (see Table 1).

**Table 1.** SHP intervention components.

SHP component	Details
1. Healthcare worker education programme	For community health workers known as ASHA’s, and primary care doctors.
2. SMARThealth Pregnancy (SHP) app	Contains a clinical decision-support algorithm to support the screening, referral and management of anaemia, hypertension, and diabetes.
3. SMARThealth Pregnancy dashboard	Provides analytics, oversight and tracking.
4. Supply chain and logistics support	Monitored via the SHP app and feed back to PHCs and district officials.
5. Stakeholder engagement	With community stakeholders to increase awareness of the programme and high-risk pregnancy conditions, and with primary care doctors and district officials to ensure the programme relevance for local priority needs and community ownership.

SHP: SMARThealth Pregnancy; ASHA: Accredited Social Health Activist; PHC: Primary Health Centre.

## The SHP trial

The SHP trial is a pragmatic, type 2 hybrid effectiveness and implementation, parallel-group cluster randomised trial across two states in India (Haryana and Telangana). The trial aims to improve the community-level identification, diagnosis, referral, follow-up and management of women with anaemia, diabetes, and hypertension during pregnancy and in the year after birth.^26^ Specific objectives of SHP are to (i) determine if the SHP intervention can improve women’s health in the year after pregnancy, specifically by decreasing the prevalence of anaemia by 9%; (ii) to determine if SHP can improve follow-up care for women with GDM or HDP; and (iii) to identify the key areas to support implementation of the intervention beyond the trial phase.^26^ Recruitment for the SHP trial started on 4 June 2022, and was completed on 19 December 2023. The last date of participant follow-up is scheduled for 15 April 2025. The full trial protocol, including a detailed logic model, of the SHP complex intervention has been published elsewhere.^26^

### Process and implementation evaluation

Various mechanisms and processes contribute to effectiveness in a complex intervention. Process evaluations accompanying RCTs help to evaluate the effectiveness of an intervention by understanding how it was implemented, the mechanisms by which it achieved the effect, and in which ways the intervention interacted with the context in which it was implemented.^27,28^ An implementation evaluation, on the other hand, aims to understand how the different components of an intervention were adopted, followed, and sustained, providing insights into post-trial sustainability and adoption.^29^

For the SHP trial outlined above, a process and implementation evaluation will be conducted alongside the trial. In order to capture both process and implementation outcomes and to understand how the SHP intervention has been embedded and normalised in practice, a combination of frameworks will be applied. We will utilise the Medical Research Council (MRC) guidance,^30^ Realist evaluation,^31^ normalisation process theory (NPT),^32^ the reach, effectiveness, adoption, implementation and maintenance (RE-AIM) framework,^33^ and Proctor’s typology^34^ to understand how the intervention was delivered and implemented and to inform strategies for its broader adoption and long-term sustainability.

### Aims

Through evaluation of the process and implementation of the SHP trial, we aim to (i) assess whether the intervention was delivered as designed and (ii) understand how the SHP intervention was implemented. This evaluation will help clarify assumptions around causal mechanisms, and enhance understanding about the generalisability of the intervention components for future implementation. The specific objectives of this process evaluation are to:

1. Assess whether the SHP trial has been implemented as intended, focusing on the degree of adherence to the planned protocol (fidelity) and the extent to which the intervention was delivered (dose).
2. Evaluate how, why, and to what extent the SHP intervention has been effective considering factors such as RE-AIM.
3. Identify and analyse the barriers, facilitators, and strategies that influenced the implementation of the SHP intervention.
4. Understand the necessary conditions and strategies required for the SHP intervention to be sustained and scaled up in clinical settings, in contexts similar to the trial sites.
5. Understand how contextual factors and mechanisms as well as unexpected consequences impacted the trial outcomes and the overall success of the intervention.

## Methods

### Study setting

The study will be community-based and conducted in rural and semi-rural districts in India: Siddipet district in Telangana State and Jhajjar and Rohtak districts in Haryana State, India. Across India, the maternal mortality rate has significantly dropped in the last decades to an average 113 per 100 000 live births in 2018, according to estimates from the Sample Registration System (SRS).^35^ Recent estimates from the Health Management Information System (HMIS) indicate that maternal mortality in Telangana has dropped to 53 maternal deaths per 100,000 live births in 2017–2019^35^ and has been maintained at less than 70 in 2019–2021.^36^ In comparison, depending on estimates, Haryana had a higher maternal mortality rate of 101-91 for 2014–2018 (SRS) and 90 for 2017–2019 (HMIS), for 214 maternal deaths per 100 000 live births before 2018.^37^ As reported in the recent Comptroller and Auditor General of India’s Performance Audit, contrary to national rates, MMR in Haryana has increased from 98 (2015–2017) to 110 (2018–2020).^38^

The study will be conducted in both rural and semi-rural areas of India, specifically in the Siddipet district of Telangana and the Jhajjar and Rohtak districts of Haryana. These locations were strategically chosen to represent two distinct geographical, linguistic, and cultural populations. Each of the selected PHCs serves approximately 30,000 people across multiple villages. From each PHC’s catchment area, two villages will be selected to serve as the study sites for the trial. All community health workers and PHC staff from these sites will be eligible to participate in the study.

### Study design

A sequential, mixed methods study is designed to assess each of the objectives of the process evaluation. The process evaluation will run concurrently with the SHP trial, which will include both the intervention and active control arms. The Standards for Reporting Implementation Studies (StaRI) checklist^39^ was utilised supporting transparent and accurate reporting of this process and implementation evaluation (Supplemental Material).

### Theoretical frameworks

The process and implementation evaluations of the SHP trial are informed by a number of theoretical frameworks adopting a pragmatic approach to support the translation of research evidence into practice. As no individual framework met the different requirements of the implementation and process analysis, a number of frameworks were combined to guide the analysis. These frameworks provide structure and insight into the complex nature of the intervention and its components.

### Integration of frameworks in data collection and analysis

To avoid conceptual overlap, the frameworks are applied sequentially and hierarchically, with clearly delineated roles across study phases. The MRC framework provides the overarching structure for the process evaluation, defining the domains of implementation, mechanisms of impact, and context. Within this structure, RE-AIM and Proctor/Weiner constructs are used operationally to guide data collection and descriptive analysis of implementation outcomes (e.g. reach, fidelity, acceptability, and sustainability). Realist Evaluation underpins theory-driven analysis by linking these implementation outcomes to context–mechanism–outcome (CMO) configurations, enabling explanation of how and why outcomes occur across settings. NPT is applied analytically to selected qualitative data to explain mechanisms related to intervention embedding and routinisation. Together, the frameworks are not used in parallel but are integrated across stages of data collection, analysis, and interpretation, ensuring theoretical complementarity rather than redundancy.

The overall development and evaluation of the intervention follow the U.K. MRC guidance on implementation and evaluation of complex interventions,^40^ using theories in order to understand and explain the process, components, and strategies of the intervention, which may or may not have been effective.^41^ The MRC framework highlights the need to unveil causal assumptions and defines three broad areas for evaluation: implementation (what is implemented, and how), mechanism of impact (how the intervention leads to change), and context (how context affects implementation and outcomes).^40^ This process evaluation has been conceptually designed along those three areas.

Within this overarching structure, the implementation evaluation will be guided by the RE-AIM framework, an established and widely used framework for planning and evaluating health interventions.^33^ The RE-AIM framework focuses on five implementation dimensions: reach, effectiveness, adoption, implementation fidelity, and maintenance.^42^ In order to assess acceptability and sustainability of the implementation, Proctor’s typology^34^ and Weiner’s implementation measures^43^ will also be applied.

A realist evaluation approach will be used at the analytical stage to test the initial programme theory (process evaluation framework) to explain how the *context and mechanisms* of the SHP intervention work, for whom, and under which circumstances.^31^ In the context of complex interventions and RCTs, such as SHP trial, realist evaluation is an effective method for understanding and clarifying key components.^36^ Realist evaluation focuses on the interplay between CMOs, allowing interpretation of observed implementation outcomes across different settings and participant groups. This evaluation method will help clarify how the intervention produces both intended and unintended effects, as well as the contextual factors that contribute to its success or failure.

In addition, NPT will be used as a focused analytical lens to examine how the SHP intervention becomes embedded in the routine practices of healthcare workers and communities. NPT is widely used to identify, characterise, and explain mechanisms that affect the implementation of new health processes, technologies, and other complex interventions.^44^ It focuses on four key constructs: coherence (e.g. what is the purpose of the SHP intervention), cognitive participation (what promotes participation in the SHP intervention), collective action (how do women and ASHAs interact with the SHP app to make them work for them), and reflexive monitoring (how do ASHAs, women and PCPs, appraise the intervention’s effectiveness).^32^ NPT will be applied to selected qualitative data to elucidate mechanisms related to participation, enactment, and appraisal rather than to assess implementation outcomes per se.

Table 2 summarises the purpose and application of frameworks applied.

**Table 2.** Overview of frameworks applied to the SHP study.

Framework/theory	Purpose	Application
UK MRC guidance on implementation and evaluation of complex interventions^40^	To understand and explain the process, components, and strategies of the intervention to unveil causal assumptions	Overall SHP study implementation (what is implemented, and how), mechanism of impact (how the intervention leads to change), and context (how context affects implementation and outcomes)
RE-AIM framework^33^	To test the initial programme theory (process evaluation framework) To understand how the *context and mechanisms* of the SHP intervention work, for whom, under which circumstances	Implementation evaluation of the SHP intervention: reach, effectiveness, adoption, implementation fidelity, and maintenance
Proctor’s typology^34^ and Weiner’s implementation measures^43^	To assess acceptability and sustainability of the implementation	Implementation evaluation of the SHP intervention: Acceptability, sustainability
Realist evaluation	To test the initial programme theory (process evaluation framework) and to understand how the *context and mechanisms* of the SHP intervention work, for whom, and under which circumstances	Applied to the programme theory of the overall SHP study: context, mechanisms, and outcomes
NPT^44^	To analyse and explain how the SHP intervention becomes embedded in the routine practices of healthcare workers and communities	Applied to understand the embeddedness of the SHP intervention into routine practices

MRC: Medical Research Council; NPT: normalisation process theory; SHP: SMARThealth Pregnancy; RE-AIM: reach, effectiveness, adoption, implementation and maintenance.

**Figure 1.**
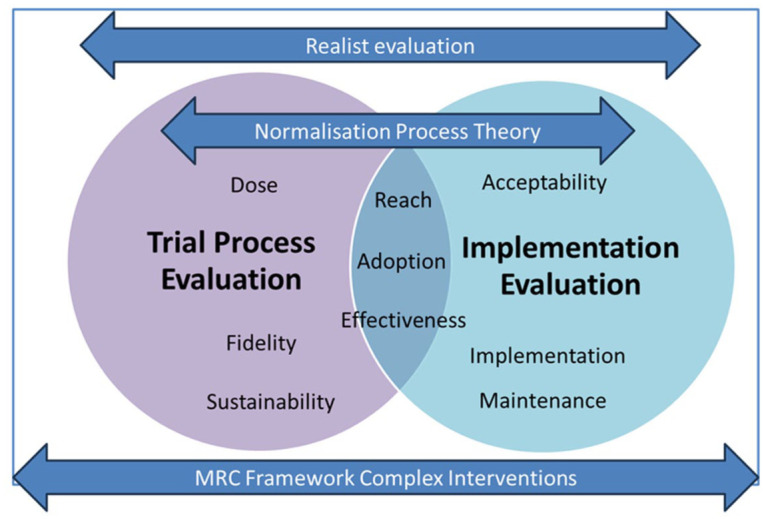
Integrated conceptual approach to the SHP2 process and implementation evaluation. SHP: SMARThealth Pregnancy.

Figure 1 outlines how the different theories and frameworks are being combined.

The design and reporting of the process evaluation will adhere to the Quality Standards for Realist Evaluation^45^ and the StaRI checklist.^46^

### Process evaluation framework

**Figure 2.**
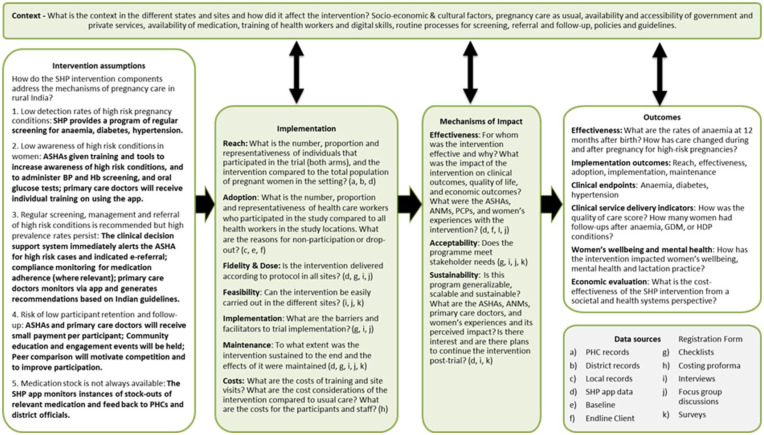
The SHP process evaluation framework. SHP: SMARThealth Pregnancy.

The process evaluation framework (initial programme theory) outlines how and under which conditions the SHP intervention is expected to lead to its desired effects (see Figure 2). Specifically, the framework helps to capture how we expect the SHP intervention in rural India to work for the stakeholders (women, ASHAs, primary care doctors), how the context may influence the implementation, mechanisms, and outcomes, and how and which components of the implementation and mechanisms are leading to the expected outcomes, and contribute to the overall aim of improving women’s health outcomes in the perinatal phase, for conditions like anaemia, diabetes, and hypertension in rural India.

The process evaluation components (highlighted in green) are exploring contextual factors, the implementation of the SHP trial, and mechanisms of impact from the intervention. Questions were developed based on the RE-AIM and Realist frameworks to fit within these components. The causal assumptions of the SHP intervention inform the process evaluation components and the interpretation of the primary and secondary outcomes.

During the process evaluation, we will continue to refine the overall SHP programme theory, as well as the assumptions about how the intervention works, and develop implementation strategies, to address barriers and use facilitators to implementation. These strategies will be derived from the data evaluation, fidelity checklists, and oral/written reports, as well as iteratively informed by a continuous feedback loop from end users (women) and implementers (ASHAs/primary care doctors/field supervisors) on what works (and what does not), and any strategies applied will be captured in a log.

**Figure 3.**
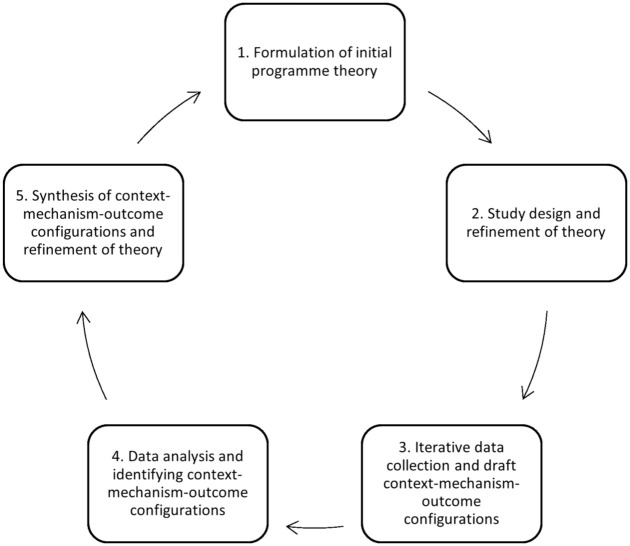
Analysis phases applied in the SHP2 realist evaluation. SHP: SMARThealth Pregnancy.

Figure 3 provides an overview of the analysis phases planned in our realist evaluation.

### Implementation strategies

During the trial, a number of strategies will be applied to support implementation success and overcome any barriers that may arise.^26^ The implementation strategies were designed based on findings of the pilot trial,^25^ in consultation with the study team and local experts, and informed by the Expert Recommendations for Implementing Change (ERIC) compendium.^47^ The ERIC compendium is a list of discrete strategies that can serve as building blocks for constructing multifaceted, multilevel implementation strategies. Our identified strategies for SHP trial include (i) strategies to improve the implementation process (e.g. staff support for ASHAs), (ii) strategies to improve adherence to the intervention (e.g. regular quality control), (iii) strategies to build capacity (e.g. through regular refresher training), or (iv) strategies for dissemination (e.g. community events). A list of planned implementation approaches for each of these strategies is shown in Table 3, which was developed considering the recommendations for specifying and reporting implementation strategies by Proctor et al.^48^ From the start of the SHP study, the strategies will be continuously reviewed and adapted as needed. The process will ensure that any identified barriers to implementation are addressed promptly and effectively. Strategies and any modifications will be documented throughout in a log.

**Table 3.** Planned strategies to support implementation of the SHP trial (intervention arm).

Category	Target	Implementation strategy/action	Temporality	Dose	Implementation outcome targeted
I. Strategies to improve the implementation process	Women	Information collected from health system on mortality and morbidity of pregnant mothers and their newborn. This information collected by study team and will help in addressing the major drivers of maternal and newborn mortality. This will help achieve successful programme implementation and outcomes.	Periodic	Regularly	Implementation
		For women who move back to their mother’s village for the baby’s birth visits will be replaced with phone calls, resuming face-to-face visits when the woman returns to her original village.	Continuous	Over the period of the woman’s stay at her mother’s house	Effectiveness, adoption, implementation, maintenance
	ASHAs	Study staff will support ASHAs by attending visits (with participant permission) and providing support for using the technology until the ASHA feels confident of performing assessments independently.	Continuous	Initially for the visits, then as needed/identified in the fidelity checklist	Effectiveness, fidelity, adoption, acceptability, implementation
	Women, ASHAs, primary care doctors, district officials, community, field staff	Continuous feedback loops to enable corrections and changes that are necessary to achieve successful programme implementation and outcomes	Continuous	Regularly	Effectiveness, fidelity, adoption, acceptability, implementation, maintenance
II. Strategies to improve adherence to the intervention	ASHAs	Quality control visits will be made to observe the intervention delivered by ASHAs and assessed by the study team.	Periodic	1 visit	Fidelity, effectiveness
		Compliance of the ASHAs with the intervention activities will be assessed with a fidelity checklist, administered by the study team	Before and after refresher training	Twice	Fidelity
	ASHA, primary care doctors	Compliance with the intervention visits will be monitored centrally by tracking the visits recorded in the SHP app. If trends of non-engagement across multiple participants from the same ASHA worker or PHC are noted, the study team will arrange a site visit.	Continuous	Regularly	Fidelity
III. Strategies to build capacity	ASHAs	Every 6 months, the study team will conduct a 1-day refresher training session for the ASHAs.	One day every 6 months	1 day	Reach, effectiveness, implementation
IV. Strategies for dissemination	Community	Community stakeholder engagement events will be held to increase awareness of the programme and the importance of high-risk pregnancy conditions, and co-develop pathways to implementation and sustainability after completion of the trial.	Before start, and after completion of the trial	Regularly	Implementation, maintenance, acceptability
	Women	Stakeholder engagement events will be held to increase awareness of the programme and the importance of high-risk pregnancy conditions, and co-develop pathways to implementation and sustainability after completion of the trial.	Before start, during, and after completion of the trial	Regularly	Implementation, maintenance, acceptability
	District officials, primary care doctors	We will regularly update and engage with district officials and primary care doctors to ensure the programme addresses local priority needs and is seen as being owned by the communities.	Continuous	Regularly	Implementation, maintenance, acceptability

SHP: SMARThealth Pregnancy; ASHA: Accredited Social Health Activist; PHC: Primary Health Centre.

Throughout the project we will be applying responsive feedback loops, an approach that is effective in providing agile, flexible, adaptive, iterative, and actionable feedback.^49^ Feedback loops provide timely assessments and actionable feedback by creating a communication circle between the project leads, implementers, researchers, healthcare workers, and decision-makers which enable corrections and changes that are necessary to achieve successful programme implementation and outcomes. Responsive feedback loops have more recently also been incorporated in process evaluations of RCTs using digital interventions.^50^

### Participants

The participants for the qualitative components of this process and implementation evaluation, including focus group discussions (FGDs) and in-depth interviews (IDIs), will include a range of individuals involved in the SHP intervention. Both FGDs and IDIs are used to capture the perspectives of different stakeholders. FGDs will be conducted with ASHAs who participate in the training and are using the SHP app and diagnostic devices during their visits to explore both their shared and individual experiences in balancing their existing routine responsibilities and SHP activities, as well as their interactions with primary care doctors, auxiliary nurse midwives (ANMs), and women. IDIs will be conducted with women, primary care doctors, ANMs, health officials, women, and field supervisors in order to provide a space for participants to share sensitive details, personal reflections, and a secure environment to discuss their experiences (women). In addition, practical considerations around feasibility determined the mixed use of FGDs and interviews.

Sampling will be guided by thematic saturation, defined as the point at which no new themes or relevant insights emerge from successive interviews or FGDs. Based on the planned study design and practical feasibility, qualitative data collection will be conducted in 2 rounds (midline and endline), with an anticipated total of 32 IDIs and 12 FGDs across both field sites, as detailed in Table 3 (see below, under qualitative data collection plan). This will include 8 IDIs with pregnant and postpartum women, 8 IDIs with primary care doctors, 4 IDIs with ANMs, 8 IDIs with field supervisors, 4 IDIs with policy or health officials, and 12 FGDs with ASHAs. Final sample sizes may vary depending on achievement of thematic saturation.

Participants will be selected from those already involved in the SHP2 trial. Policy makers will be identified with the assistance of the district and state administrative heads. For healthcare workers, all healthcare workers who participated in the SHP study were eligible. For women, all participants of the study are eligible to participate, regardless of whether they have completed all assessments or not. In addition, women who have rejected participation are eligible. Participants who decline participation in the process evaluation are ineligible.

The study team will also identify independent experts from these areas to facilitate qualitative interviews. These interviews and FGDs will be conducted at convenient locations for participants. All sessions will be audio-recorded, with informed consent of the participants.

### Informed consent process

Participants will provide consent before qualitative interviews or focus groups by personally signing the Informed Consent Form (ICF) after reviewing the participant information sheet. Both documents, outlining the interview process, participant role, and any potential risks, will be available in written and verbal formats in the local language.

The consent process will be managed by a qualified and experienced member of the research team, who will be authorised by the Chief Investigator. A copy of the signed ICF will be given to the participant. The original signed form will be retained at the study site. Also, where desirable and acceptable to participant groups, a summary of the discussion proceedings will be written and countersigned by all participating in the discussion meeting. The data collected during these interviews and focus groups will be handled in accordance with the data protection policy for the SHP trial. All personal and sensitive information will be kept confidential and stored securely to protect participants’ privacy. The policy will also ensure that participants’ rights are respected, including their ability to withdraw from the study at any point without penalty.

During data collection, all study participants will be allocated a unique study identifier. No personal data (names, addresses, contact details) will be stored in the study database. A password protected database containing personal information needed for participant follow-up will be maintained for use by designated study field investigators only for the duration of the period of participation of an individual in the study. All information collected on participants will be kept confidential and used only for the purposes outlined in the ICF by study staff and clinicians involved in the study.

### Data collection

The data collection for this mixed methods study includes both quantitative and qualitative data, collected from a variety of sources such as SHP app analytics, trial data, interviews and FGDs.

Assessment measures and planned forms of measurement has been previously published in the protocol for the SHP main trial.^26^

The sample size for this process and implementation evaluation is determined by the objectives of the evaluation, the types of data to be collected (quantitative and qualitative), and practical considerations including resources and time. For the quantitative study, we aimed to include either the full sample, and/or from all staff and a representative sample of women. For the qualitative evaluation, we included a smaller, more purposive sample.

### Quantitative data collection

Quantitative data will be sourced from the SHP trial database.^26^ Data will be extracted from the SHP app, which tracks ASHAs’ screening and follow-up activities and the number of primary care doctor visits. At the intervention sites, the ASHA worker will conduct a health assessment of the women during home visits, gather data on pregnancy, childbirth, and treatment (if any) received during or after childbirth, and enter this data in the SHP application. Data will be collected in the SHP app on the consumption of iron and folic acid tablets for improving blood haemoglobin, health examinations performed, haemoglobin levels, and blood pressure readings, as well as any other specific diseases or treatments received during pregnancy and after delivery. This information will help understand how the health status of the women has changed during the project’s duration when compared to women receiving usual care in the control sites. In addition, quantitative data from trial records, district records, surveys, checklists, and costing proforma will be collected, as outlined in the SHP trial protocol paper.^26^

### Qualitative data collection

The process evaluation framework will guide the qualitative data collection. Data collection will be done using pre-/ mid-/ post-implementation questionnaires in two rounds: round 1 (planned April to May 2023 and September to December 2023) and round 2 (planned November 2024 to January 2025), audio analysis of interviews and focus groups, observation of daily routines and interactions, fidelity checklists, journey mapping, and diary/project log analysis.

IDIs and FGDs will be conducted with representatives from beneficiaries (women participating in SHP trial), healthcare providers (ASHAs, ANMs, medical officers, healthcare providers), and field supervisors/staff in each field sites involved in implementing and influencing policy decisions concerning maternal and child health and NCDs in the state. A representative sample of participants from the study sites and clusters will be purposely selected to capture differences in PHC qualities. The questionnaires will be developed using the RE-AIM framework and Proctor et al.’s implementation outcomes.^34^ Interviews and focus groups will be conducted in Telugu/Hindi, audio-recorded, transcribed verbatim in the original language, and translated into English for analysis. To ensure accuracy and preserve meaning, a subset of transcripts will be independently reviewed against the original recordings, and discrepancies will be resolved through discussion within the research team. Key terms and culturally specific expressions will be discussed among the team and meanings verified with bilingual speakers and the data collectors to ensure conceptual equivalence.

Additional qualitative methods applied will be observation and patient journey mapping of high-risk women to understand how they enter, navigate, experience, and exit the SHP programme. Furthermore, we will explore their experiences within existing private and public healthcare. These methods will capture patient behaviours, feelings, motivations, and attitudes across care episodes.

Data collection was designed according to the objectives of the process evaluation. To understand how and why the SHP intervention may be effective, a relatively larger representation from healthcare providers is planned, as these are directly involved in using and delivering the SHP trial components. To understand for whom the implementation is useful, IDIs with women as the end beneficiaries will be conducted, and to gain in-depth accounts of their experiences. The number of IDIs and FGDs is also guided by feasibility. It is expected that in particular the interviews will provide a rich qualitative understanding and will complement the larger quantitative data of end beneficiaries conducted in the trial.

An iterative approach to data collection and analysis will be used until data saturation (i.e. when no new themes are emerging). Throughout the trial, the research team will regularly organise meetings with stakeholders and debriefing sessions with field supervisors to review emerging findings. If needed, additional interviews will be conducted.

An overview of the overall data collection plan for the SHP process evaluation is shown in Table 4.

**Table 4.** Overview of process evaluation data collection plan.

Stakeholders (intervention group)	*n* IDIs/FGDs in both fieldsites	*n* IDIs/FGDs in both fieldsites	
	Round 1: 12 months	Round 2: endline	Total *n* IDIs/FGDs
Women			
Pregnant and postpartum	4 IDIs	4 IDIs	8 IDIs
Healthcare providers			
ASHAs	6 FGDs	6 FGDs	12 FGDs
Primary care doctors	4 IDIs	4 IDIs	8 IDIs
ANMs	4 IDIs	0	4 IDIs
Project field team/managing the supply chain			
Field supervisors	4 IDIs	4 IDIs	8 IDIs
Policy officials			
Healthcare officials	0	4 IDIs	4 IDIs
Total intervention	16 IDIs and 6 FGDs	16 IDIs and 6 FGDs	32 IDIs and 12FGDs

ASHA: Accredited Social Health Activist; FGD: focus group discussion; IDI: in-depth interview; ANM: auxiliary nurse midwife.

### Process data collection

In addition to data gathered from interviews and SHP electronic questionnaire, a range of process data will be gathered through other mechanisms to capture additional context and individual-level information that might influence the study outcomes. Project supervisors and project managers will maintain a diary to gather information about the different activities, events or interventions by the government or other agencies in the study sites that can influence the study outcomes. In addition to this health system level data, the field team will also collect and record individual level information about the participants which is not captured by the SHP application; for example, any special diet taken by the women to improve their blood haemoglobin during, after pregnancy or details of treatment received during pregnancy or postpartum due to maternal or child complication, any socio-economic factor that can influence the general well-being of the women participating in the study.

### Mixed methods data collection for process and implementation evaluation

#### To assess whether the SHP study has been implemented as planned (implementation fidelity, dose)

To assess whether, and to what extent, the study has been implemented as intended, we will assess fidelity and dose (number of visits) of the trial procedures themselves. This will be collected using process data records, including frequency of training, completion of electronic case record files, and activity logs, drawing on SHP app-based data sources described above. Explain whether, how, and why the intervention has been effective (Reach, Effectiveness, Adoption, Acceptability, Implementation, Maintenance).

We will collect effectiveness and implementation outcomes.

- Effectiveness outcomes: This will be based on the primary and secondary outcomes of the SHP trial.^26^
- Implementation outcomes: This will be assessed using the RE-AIM framework and Proctor et al.’s typology. Reach will assess the potential reach of the SHP intervention, that is, whether it is used in all eligible participants (proportion of the target population and its representativeness). Effectiveness will assess the impact on health outcomes, including positive, negative, and unintended consequences. Adoption will assess how well and to what extent the intervention has been taken up by the participants of the SHP trial (including how representative this is for the target population in the setting). Implementation will explore the extent to which each of the SHP components were implemented and/or adapted, including an assessment of the costs of delivery. Maintenance will assess the degree to which SHP was sustained at both setting level (in sites) and individual level (among participants). Acceptability will assess to what degree the intervention was acceptable for the participants of the trial (women, ASHAs, primary care doctors). Feasibility will assess to what extent the SHP intervention can be adopted, implemented, and sustained as intended. To assess this, a range of data will be used, including trial data, checklists, surveys, and costing and administrative data, with app-based process data drawn from the SHP platform as described above. Topic guides for qualitative interviews and focus groups will be informed by the process evaluation framework, Proctors’ framework^34^ and Weiner’s Implementation Measures,^43^ translated into local languages, and piloted before administration.

#### Examine how barriers, facilitators, and applied strategies affected the implementation of the intervention

To understand the impact of barriers, facilitators, and applied strategies on the implementation process, qualitative methods (interviews, FGDs and surveys) will be conducted during and after the implementation of the study. The data collection will explore perceptions of barriers and facilitators of the intervention, by women, ASHAs, ANMs, primary care doctors, district officials, and study team. In addition, data from the process evaluation framework on maintenance and sustainability (see above) will feed into the analysis.

Throughout the trial implementation and evaluation process, we will incorporate feedback loops to assess the effectiveness of the strategies, and to improve the quality of the process implementation. Regular quantitative and qualitative monitoring data from field supervisor reports/notes, logistics chain surveillance, and mid-line assessments, together with SHP app-based monitoring data described above will be triangulated and revised. Additional intervention strategies will be defined by the operations group and the delivery team to ensure implementation success.

#### Understand what and how contextual factors and mechanisms impacted the outcomes

The realist evaluation approach will be used to understand contextual factors and mechanisms that contributed to the implementation and outcomes. As defined by the realist approach, in order to analyse the effectiveness of an intervention, it is essential to understand mechanisms (M) and the contexts in which they operate (C) and how they affect an outcome (O) (C + M = O).^31^ Assumed mechanisms will be tested and refined through both quantitative data and qualitative insights from interviews and FGDs. Stakeholder input will be essential in developing these configurations and refining the programme theory.

#### Understand what might be required for SHP to be sustained and widely implemented in similar clinical settings (sustainability)

Qualitative interviews and FGDs will be conducted during and after the implementation of the study to better understand what may be required to sustain the intervention. The data collection will explore perceptions of the intervention, and any barriers or opportunities to wider implementation by women, ASHAs, ANMs, primary care doctors, district officials, and study team. In addition, perspectives of decision-makers in government will be consulted to explore financial and technical sustainability and policy implementation, including early insights to integration to existing platforms or other opportunities moving from project to programme which would be fully run and administered by the local authority. In order to understand the extent to which the intervention can be maintained and continued within an ongoing service setting, including its durability, we will administer the Program Sustainability Assessment Tool^51^ with women, ASHAs, ANMs, primary care doctors, district officials, and study team after completion of the trial.

### Data analysis

The process evaluation will be analysed using a mixed methods approach. The analysis will aim to seek possible explanations for implementation and process outcomes achieved in the SHP trial.

Quantitative and qualitative data will be triangulated and reported together to provide a holistic understanding of the implementation process and outcomes. Data from multiple stakeholders namely women, ASHAs, ANMs, primary care doctors, district officials, and study team will be triangulated to ensure that multiple perspectives are captured. The process evaluation framework (Figure 2) will guide the data analysis for process evaluation data. This framework will ensure that all aspects of the intervention, context, mechanisms, and outcomes are analysed comprehensively.

The quantitative data will be analysed as part of the overall SHP trial statistical analysis plan and will be published online before the SHP database will be locked. Contextual variables captured through process data (e.g. health system factors, implementation intensity, site-level characteristics, and participant socio-demographic factors) will be operationalised as covariates or stratification variables in the quantitative analysis. Where appropriate, multivariable regression models and subgroup analyses will be used to assess the influence of contextual factors on primary and secondary outcomes. Qualitative exploration through interviews and FGDs will inform interpretation of all major process and implementation themes.

**Figure 4.**
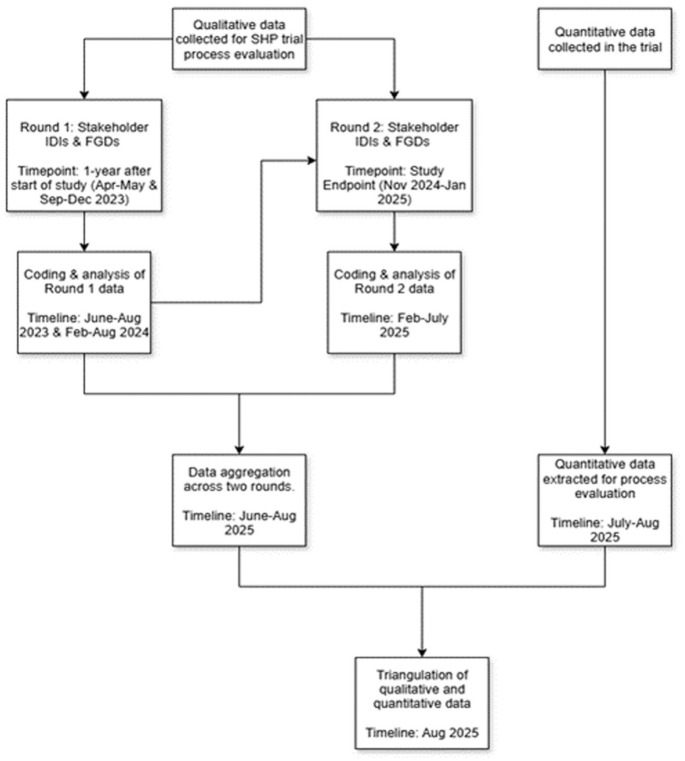
Flowchart of data collection, analysis, and triangulation points.

Qualitative data analysis will be done in several stages, using a mixed deductive and inductive thematic analysis.^45^ Audio recorded data from interviews and FGDs will be translated and transcribed in English. Thematic analysis of the data will be conducted using Atlas.ti software. Coding will be conducted by two trained qualitative researchers from the study team. An initial subset of transcripts will be independently coded to develop and refine the coding framework. Inter-coder agreement will be discussed and discrepancies resolved through consensus, with iterative refinement of the codebook before coding of the full dataset. Data will be coded using the constructs of the process evaluation framework (Figure 2, above). A combination of deductive and inductive coding will allow for generation of new themes outside these frameworks. Themes will be generated, and subsequently common and distinctive key themes and constructs will be identified based on frequency (e.g. elicited in >60% participants) and expressed relevance of participant groups (patients, healthcare workers, primary care doctors, field staff). The themes and findings will be triangulated with findings from observations, patient journey mapping, surveys, notes from field staff and SHP app data, and other information. Reporting of qualitative data will follow the COREQ guidelines.^52^ To investigate the influence of context and mechanisms on outcomes, the findings from the CMO configurations will be analysed using thematic analysis using Atlas.ti, descriptive statistics, and multivariate analysis of variance to assess differences in mechanisms. Figure 4 outlines the flow of data collection, analysis, and triangulation points.

### Ethical approval

The SHP trial received ethical approval from OXTREC (reference 27-21) on 24 November 2021, and the George Institute for Global Health ethics committee (reference 22/2021) on 1 December 2021, with version 2.0 approved in November 2022. Version 2.0 included details on the process analysis and the addition of a mid-line study visit in both study arms to facilitate accurate collection of birth outcomes. Written informed consent to participate will be obtained from all participants (PHCs, health workers, and individual women) prior to commencement of study. Women will be free to withdraw their consent at any time during the study without affecting their access to services.

To protect confidentiality in small rural communities where anonymity may be challenging, all data will be de-identified at the point of transcription and analysis, removing personal identifiers and using unique study codes. Access to identifiable data will be restricted to authorised members of the research team, and findings will be reported in aggregate or using anonymised quotations that avoid potentially identifying details (e.g. specific locations or roles).

In the event that incidental findings or medical emergencies are identified during study visits or interactions, participants will be informed accordingly and referred to appropriate local health services following standard clinical and referral protocols. Where required, study staff will notify supervising clinicians or local health authorities to ensure timely follow-up, in line with ethical approvals and participant consent.

### Patient and public involvement

In the earlier pilot study, community feedback was collected through FGDs and IDIs. During the main SHP trial, continuous engagement with trial participants and other relevant stakeholders is being maintained. The SHP process evaluation study findings will be shared with the public. Findings will be disseminated through publication in peer-reviewed journals, meetings, digital and social media platforms.

### Trial status

The SHP trial’s first patient was recruited in June 2021 and recruitment of patients exceeded the required sample size of 3240 in December 2023. The trial is expected to conclude in April 2025. The SHP process evaluation data collection started in June 2022 with observational visits in the sites, interviews and FGDs started in November 2022, the fidelity checklist has been administered in August 2024. Data collection will be ongoing until the follow-up, and endline data collection of the main trial has been completed. Data analysis is ongoing in iterative stages, with preliminary findings supporting the intervention strategies.

## Conclusion

This process evaluation is an integral component of the SHP hybrid type-2 effectiveness-implementation trial and will be conducted concurrently with the main SHP trial. It aims to provide valuable and actionable insights into the implementation outcomes, underlying mechanisms, and contextual factors, as well as the barriers and facilitators influencing the intervention delivery. These insights will inform iterative refinement of the SHP intervention and identify the core components and implementation strategies required for successful scale-up and wider adoption.

In line with MRC guidance on process evaluations,^30^ this study will contribute empirical evidence on how complex digital and task-sharing interventions can be implemented in low-resource health systems and low- and middle-income country settings.^53^ The findings will directly inform policy-relevant questions related to feasibility, acceptability, integration within existing primary care and community health worker platforms, and resource requirements for delivery at scale. The findings from this process evaluation will provide valuable evidence for improving long-term health outcomes in perinatal women in rural India. Specifically, the results will provide decision-makers with evidence to guide adaptation of the SHP model within public health systems, inform scale-up frameworks for maternal health interventions, and support implementation planning at district and state levels. By clarifying which contextual and implementation factors are critical for sustainability, this evaluation will strengthen the translation of effective interventions into routine practice and contribute to improving maternal and perinatal health outcomes in rural India and comparable settings.

## Supplemental Material

sj-docx-1-whe-10.1177_17455057261425789 – Supplemental material for Protocol for a process and implementation evaluation of the SMARThealth pregnancy hybrid type 2 cluster randomised controlled trial Supplemental material, sj-docx-1-whe-10.1177_17455057261425789 for Protocol for a process and implementation evaluation of the SMARThealth pregnancy hybrid type 2 cluster randomised controlled trial by Nicole Votruba, Sreya Majumdar, Sudhir Thout Raj, Vaaruni Nayak, Ankita Sharma, David Peiris, Hueiming Liu, Varun Arora, Minakshi Verma, Mohammad Abdul Ameer, Devarsetty Praveen and Jane E. Hirst in Women's Health
